# The National Tumor Association Foundation (ANT): A 30 year old model of home palliative care

**DOI:** 10.1186/1472-684X-9-12

**Published:** 2010-06-08

**Authors:** Marina Casadio, Guido Biasco, Amy Abernethy, Valeria Bonazzi, Raffaella Pannuti, Franco Pannuti

**Affiliations:** 1 The National Tumor Association Foundation (ANT), Bologna, Italy; 2 Academy of Science of Palliative Medicine and "G. Prodi" Center for Cancer Research, Alma Mater Studiourm, University of Bologna, Bologna, Italy; 3 Duke University School of Medicine, Durham, N.C., USA

## Abstract

**Background:**

Models of palliative care delivery develop within a social, cultural, and political context. This paper describes the 30-year history of the National Tumor Association (ANT), a palliative care organization founded in the Italian province of Bologna, focusing on this model of home care for palliative cancer patients and on its evaluation.

**Methods:**

Data were collected from the 1986-2008 ANT archives and documents from the Emilia-Romagna Region Health Department, Italy. Outcomes of interest were changed in: number of patients served, performance status at admission (Karnofsky Performance Status score [KPS]), length of participation in the program (days of care provided), place of death (home vs. hospital/hospice), and satisfaction with care. Statistical methods included linear and quadratic regressions. A linear and a quadratic regressions were generated; the independent variable was the year, while the dependent one was the number of patients from 1986 to 2008. Two linear regressions were generated for patients died at home and in the hospital, respectively. For each regression, the R square, the unstandardized and standardized coefficients and related P-values were estimated.

**Results:**

The number of patients served by ANT has increased continuously from 131 (1986) to a cumulative total of 69,336 patients (2008), at a steady rate of approximately 121 additional patients per year and with no significant gender difference. The annual number of home visits increased from 6,357 (1985) to 904,782 (2008). More ANT patients died at home than in hospice or hospital; this proportion increased from 60% (1987) to 80% (2007). The rate of growth in the number of patients dying in hospital/hospice was approximately 40 patients/year (p < 0.01), vs. approximately 177 patients/year for patients who died at home. The percentage of patients with KPS < 40 at admission decreased from 70% (2003) to 30% (2008); the percentage of patients with KPS > 40 increased. Mean days of care for patients with KPS > 40 exceeded mean days for patients with KPS < 40 (p < 0.001). Patients and caregivers reported high satisfaction with care in each year of assessment; in 2008, among 187 interviewed caregivers, 95% judged the quality of doctors' assistance, and 91% judged the quality of nurses' assistance, to be "optimal."

**Conclusions:**

The ANT home care model of palliative care delivery has been well-received, with progressively growing numbers of patients served. It has resulted in a greater proportion of home deaths and in patients' accessing palliative care at an earlier point in the disease trajectory. Changes in ANT chronicle palliative care trends in general.

## Background

Globally, a steady increase in interest in palliative care [1,2] addresses a commitment to dignity and comfort of those with a terminal illness that transcends cultural, social and religious boundaries. Yet despite agreement on the need for palliative medicine, there is considerable uncertainty regarding the best methods for achieving palliative care objectives [3]. Among the causes for this uncertainty are inconsistencies across criteria for evaluating effectiveness, cost, feasibility, and social impact, as well as problems in the ethical, religious, legal, and cultural realms which lead to disparities in care at the end of life [4-6]. Of fundamental relevance is the question of optimal composition of the palliative care team [7]. As yet, no prospective controlled studies suggest a definitive answer to this question; assessment of seemingly identical palliative care service approaches have generated heterogeneous results.

In terms of basic structure and delivery, palliative medicine has developed three principal models: homecare, hospice care, or hospital care [8]. In any given context, the choice of a palliative care delivery model must take into account local realities, needs, and available resources. A reasoned analysis will use survey assessments and review of the established reality to match an organizational modality to the local situation [9-11]. In this article, we describe a homecare model for provision of palliative care to terminal cancer patients, a model which evolved over 30 years in Bologna, Italy, and we evaluate how this contemporary local solution matches the needs of Italians with advanced life-limiting illness.

### The team and organizational philosophy

**Figure 1:**
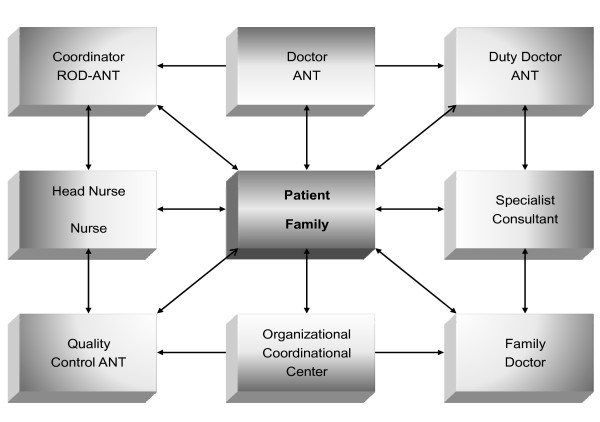
**The structure of the OHH unit (see text for details)**.

Founded in 1978, the National Tumor Association Foundation (ANT) originally operated as a volunteer association responsible for research and training in palliative care, and serving patients in the province of Bologna in the north of Italy. By 1985, ANT established the first Oncological-Hospital-at-Home (OHH), which became the basic structural unit of the organization (Fig. 1). As an operational unit, the OHH is focused on the patient and family. When a patient enrolls in the OHH program, the OHH guarantees 24-hours/day, multidisciplinary, specialized consultancy. Care is coordinated through the ANT central headquarters, which integrates the local provider within the overall social assistance process in order to ensure continuity of care. Originally made up of trained doctors and nurses, the OHH multidisciplinary team has since included psychologists (1988), nutritionists (1991) and physiotherapists (2000). The ANT staff is currently comprised of 149 physicians, 69 nurses, 23 psychologists, 64 administrative personnel, and 1,224 volunteers (Table 1).

**Table 1:** The composition of the team in the 2008

Year	2008
Physicians	149
Pharmacologists	3
Nurses & Social Workers	70
Psychologists	23
Physiotherapists	3
Administrative Personnel	64
Volunteers	1224

The OHH unit is part of the ANT palliative care network. Its board of coordinators holds responsibility for the clinical management of the patient. The board has the decisional responsibility to choose - together with the patient, family, and practitioner - the best modality of assistance throughout the clinical course, including the choice of keeping the patient at home or admitting him/her to the hospital or hospice. Doctors and nurses follow patients receiving oral chemotherapy and hormonal treatments when suggested by the referring oncologist. No infusional chemotherapy has been administered at home since 1990.

A respectful, compassionate approach to the patient and family is a key feature of ANT. The ANT staff is carefully selected and trained in order to create a sensitive but strong and trusting relationship. The objective is to create a harmonious rapport with the patient and family. Staff enter in the patients'/families' homes "on tip-toe," trying not to change the family structure and bring help without to invading interpersonal relationships and attachments. The introduction of the family into the palliative care community is gradual and generates a progressive climate of amity and trust; in this way an ANT-family interrelationship develops and becomes an essential bond within the organization [12].

From the outset, ANT has paid careful attention to the professional training of all OHH practitioners. Each staff person, before joining the care team, must have had professional training consisting of an apprenticeship period with workers in the field of palliative care and hospice. The professionalism of the ANT practitioner is updated through continuous training. ANT has invested heavily in economic and human resources to develop training for the OHH-ANT workers (doctors, psychologists, and professional nurses) in order to improve the quality of assistance for cancer patients; it organizes 10 refresher courses every year. Since 2002, the year in which the Department of Health began its experimentation for the Continuing Education in (Medicine ECM program), ANT has changed the refresher courses in accordance with the government dispositions, so as to obtain training credits for the OHH-ANT health workers. From 2002 to 2008, the ANT has provided 281 lecturers engaging 4,040 participants, for a total of 341 hours of training.

ANT activities also include diagnostic tests conducted in the home setting; these include laboratory exams, ultrasonography exams, and radiological exams to increase the usual palliative care procedures.

A possible objective index between the acquisition of scientific theory and good clinical practice can be taken from the percentage of correct answers in the tests given at the end of training events (ranging between 80 to 95%).

Finally, ANT is involved in organizing Postgraduate Courses focused to integrate home-care and hospice-care competences. These courses are organized in cooperation with the University Alma Mater Studiorum http://www.unibo.it and the Accademia of Sciences of Palliative Medicine http://www.ASMEPA.org in Bologna.

### Volunteer services

Over the years, several volunteer-provided free-of-charge health/social activities have been developed, including door-to-door services, lunch at home, clean bed service, personal cleaning of the patient, and library at home. These social activities are carried out by the volunteers, who work hand-in-hand with the professionals.

### Organization structure

**Figure 2:**
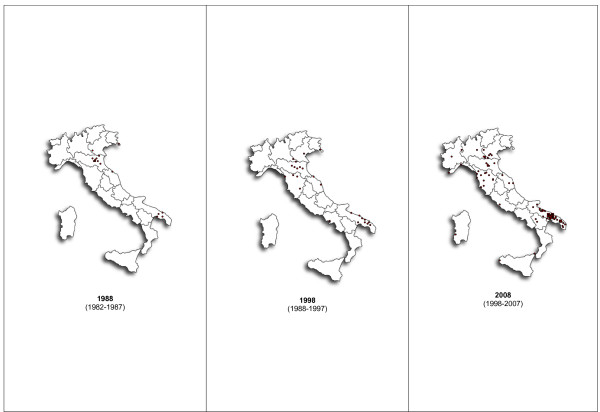
**Distribution of OHHs in Italy in the years 1988, 1988, 2008**. In 1988 the first ODO-ANT was founded in Taranto, in the south of Italy. Since that time, the number of periphery national offices has progressively increased. At the end of 2008 there were 23 OHHs in Italy.

**Figure 3:**
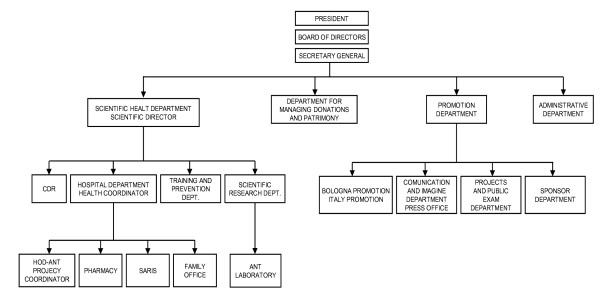
**The structure of the ANT organization (see text for details)**.

In 1988, the first ODO-ANT was founded in Taranto, in the south of Italy, and since that time, the number of periphery national offices has progressively increased. At the end of 2008, there were 23 OHHs in Italy (Fig. 2). Other structures have developed around OHHs, leading to the current organizational structure (Fig. 3). The head agency is located in Bologna and is comprised of four main departments:

1. Health department, which coordinates four activities: the data processing center, hospital-like activities, training, quality control, prevention and research. Training is organized separately for doctors, nurses, psychologists, social workers, and volunteers; activities are developed through refresher courses which provide a fundamental program for standardized up-to-date management of the patient and family. The hospital-like activity coordinates the OHHs, pharmacy, hospital-at-home ward, and family office. The pharmacy's role is to release drugs and other sanitary tools, free of charge. The family office supports the needs of the family.

2. Heredity department: many funds are devolved by patients and society. So a specific department was needed to mange and delegate use of the funds

3. Fund raising department, organized in four sections: fundraising in Bologna and in the rest of Italy; the press office; the editorial office which publishes a quarterly magazine; the project and recruitment office and the supporters' office.

4. Administrative department.

### Diffusion of activities

In addition to training courses, ANT periodically holds meetings with the public in order to introduce the local population at large to the subject of palliative care and to inform them of ANT's principles and activities.

### Development of a district network of palliative care

In 1994, the province of Bologna officially established a homecare assistance network funded by the local health system and put into effect by local practitioners. Currently, more than 200 practitioners are involved in this network, through which they work side-by-side with palliative care nurses. In 2001, the Maria Teresa Chiantore Seràgnoli Hospice with 30 rooms was opened in Bentivoglio, a small town 10 km outside of Bologna. The largest freestanding hospice structure in Italy, this organization represents an architectural and functional model for hospice-based palliative care. It is a public institution that was built with private funds. These operative structures, together with ANT, create a district network of palliative care.

### Development of a regional network of palliative care

Development of a regional approach to palliative care was advanced by the Emilia Romagna Region law of 12 January 1985, which approves a series of interventions in the territory [13]. In coordinating resources devoted to social assistance programs, this law provided for training and professional refresher courses of personnel assigned to social services, and an information system for social-assistance services that articulates with the regional information system. Despite this legislation, no modality to assist terminally ill patients was formalized, though the existing law did contain some provisions regarding social care facilities and compensation to cover relative expenses. In 1990, the public health system instituted an agreement with ANT by which it provides an economic contribution to help cover expenses of managing care for each patient; as a percentage of ANT's overall expenses, this contribution increased from 14% in 1990 to 40% in 2008.

### Funds and costs

**Figure 4:**
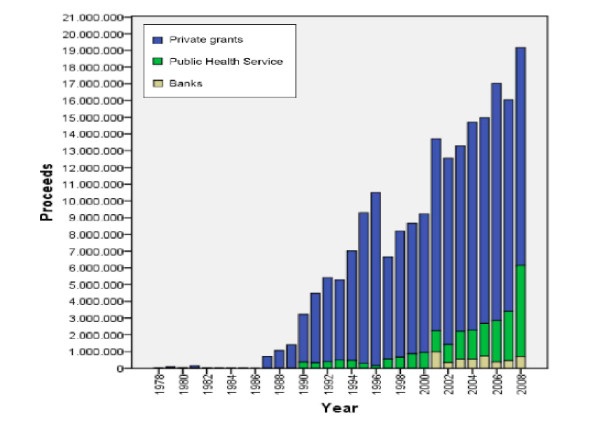
**Until 1998, ANT was financed solely with private grants**. From 1990 to 2000, contributions from bank foundations were added and in 2001, the public health system allocated funds. Private grants (blue square), Public Health Service (green square), Banks (yellow square).

ANT home assistance is provided free of charge, as it is supported by private grants and donations. Until 1998, ANT was financed solely with private grants (Fig. 4). From 1990 to 2000, contributions from bank foundations were added, and in 2001, the public health system allocated funds. The overall revenue of ANT in 2008 was 19,169,854 euros - 68% from private grants, 27% from the foundation, and 4% from the public health system. The daily cost for every patient is less than 30.00 euros, as compared with the cost for daily cost in the general hospital and in hospice of 670.00 and approximately 350.00 euros, respectively. Recently, an initiative called "Family Solidarity Project" gives economic support to families with severe financial difficulties.

### Satisfaction with care

Satisfaction with care was assessed by a questionnaire addressed to the patient or family/caregiver. Up to now only the questionnaires given to the family give available results. The questionnaires were sent to families a month after the death of the assisted spouse. Data mainly regard the province of Bologna and they refer to the year 2008. The data collected are part of a larger study which will cover all of Italy with a minimum observation period of 10 years, after which, with a separate independent note and a proper statistical study, we will give an account of the results obtained.

## Methods

All data utilized for this paper were collected from ANT archives. Statistical analysis was performed with parametric and non parametric test when appropriate.

Kyplot Version 2.0 was used to generate analyses and figures. A Linear and a quadratic regressions were generated; the independent variable was the year, while the dependent one was the total number of patients from 1986 to 2008. Further two linear regressions were generated; the independent variable was the year, while the dependent one was the number of patients from 1986 to 2008 died at home and in the hospital, respectively. The comparison between the two regressions was performed by means of a variation of the F-test, which is called the Chow test; it allows the verification of the equality between the rates of growth in the dependent variable [14]. For each regression, the R square, the unstandardized and standardized coefficients and related P-values were estimated. Additional analyses and figures focused on the proportion of patients with Karnofsky Performance Status (KPS) ≥40 and KPS < 40, length of participation in the ANT program (per patient, distinguished by group), and funding source for patient care in ANT.

## Results

### Growth and impact

**Figure 5:**
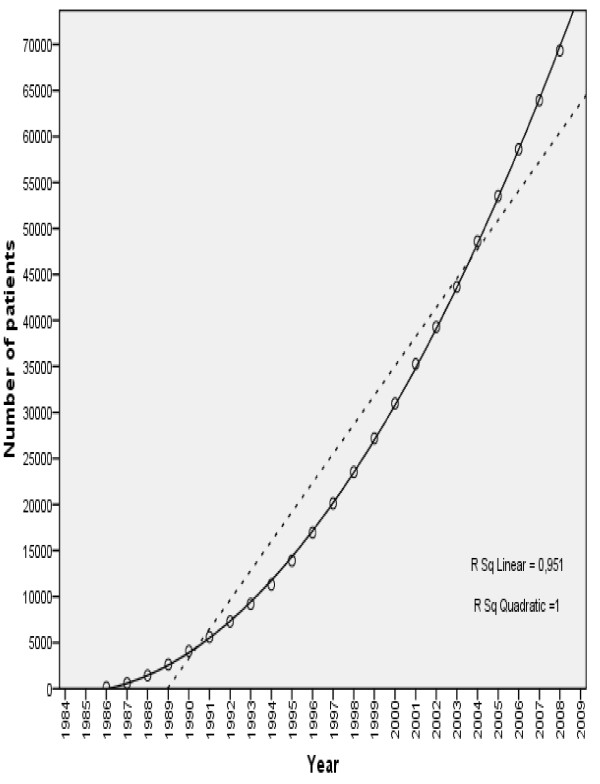
**Number of patients served by ANT from 1986 to 2008**. Over this 23-year period, the number of patients has increased with a constant acceleration of approximately 121 patients per year. The independent variable is the year, while the dependent one is the total number of patients.

The number of patients served by ANT has increased continuously from 131 in 1986 to a cumulative total of 69,336 patients by the end of 2008 (Fig.5). Over this 23-year period, the number of patients has increased with a constant acceleration of approximately 121 patients per year. There is no significant gender difference in percentage of new patients assisted. Since the mid-1990 s, the National Public Health Service has significantly increased its requests for assistance from ANT for its patients who are in terminal stages.

**Figure 6:**
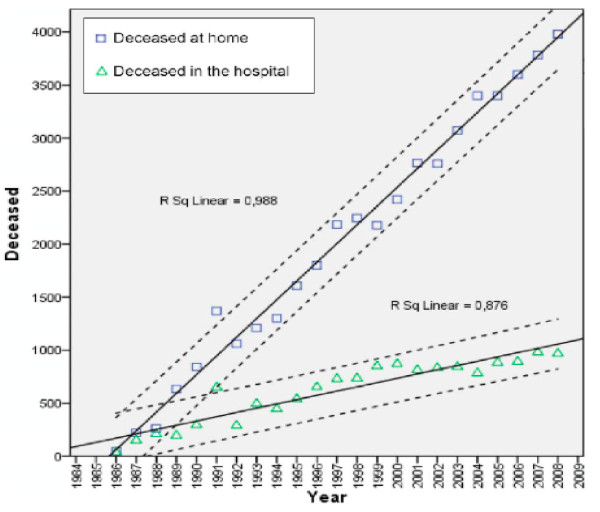
**Number of patients deceased at home (blue square), and in the hospital (green triangle) from 1986 to 2008 and the related linear regression lines**. Every year, more ANT patients died at home than died in hospice or hospital; this proportion increased from 60% in 1987 to 80% in 2007.

The annual number of home visits increased from 6,357 in 1985 to 904,782 in 2008. Every year, more ANT patients died at home than died in hospice or hospital; this proportion increased from 60% in 1987 to 80% in 2007. Analysis of the trend over this time period shows that the rate of growth in the number of patients dying in hospital/hospice is approximately 40 patients/year (p < 0.01), vs. a rate of increase of approximately 177 patients/year in patients who died at home. (Fig. 6) In 2008 the percentage of ANT assisted patients who died at home was different in every part of the country: 70% in northern, 77% in central, 90% in southern part of Italy.

**Figure 7:**
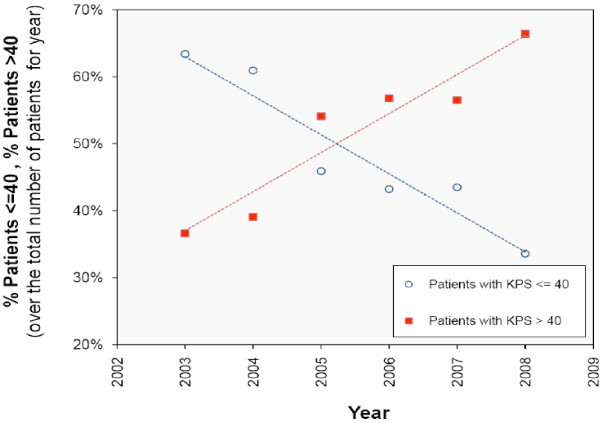
**Percentage of patients with KPS <= 40 (blue circle) and KPS > 40 (red square) from 2003 to 2008, as well as the related linear regression lines**. Change in KPS and days of care provided were evaluated. The percentage of patients with KPS < 40 at admission decreased from 70% in 2003 to 30% in 2008; the percentage of patients with KPS > 40 increased.

**Figure 8:**
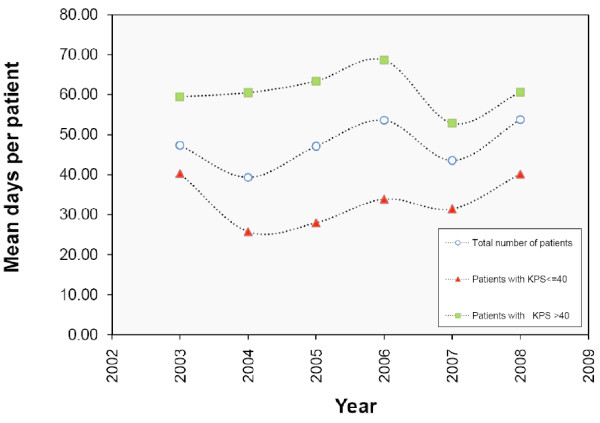
**Mean days per patient according to the performance status (KPS)**. From the years 2003 to 2008 the mean number of days devoted to the assistance is lower for patients with KPS <= 40 (red triangle) and it is higher for patients with KPS > 40 (green square) in comparison to the total number of patients (blue circle) (P. values = 0.000)

Change in performance status (KPS) and days of care provided were evaluated. The percentage of patients with KPS < 40 at admission decreased from 70% in 2003 to 30% in 2008; the percentage of patients with KPS > 40 increased (Fig. 7). Mean days of care for patients with KPS > 40 exceeded mean days for patients with KPS < 40 (p < 0.001) (fig. 8).

### Satisfaction with care

Caregivers reported a very high satisfaction with care in each year of assessment. In 2008, among 187 interviewed caregivers, 95% judged the quality of doctors' assistance, and 91% judged the quality of nurses' assistance, to be "optimal."

## Discussion

A model of palliative care strongly reflects the social-cultural conditions in which it develops. It is therefore neither feasible nor desirable to propose one single standard model for palliative care delivery and to advocate for that model across cultures around the world. While the training and basic organization of a palliative care staff share certain features to ensure quality of care, process and operational parameters must respond to the local environment in order to achieve best use of resources and optimal outcomes for patients and families.

Bologna's political system, stable for many years, has always paid a great deal of attention to social needs. In 1985, however, even in a relatively stable economic period, end-of-life care was not a primary objective of public assistance. Most cancer patients were released from the hospital without a formalized supportive care plan. ANT stepped in to address a social need that public assistance could not fully offer, and strove to do so with professional quality and at no cost to patients and families.

ANT was originally a private organization offering free-of-charge assistance. It was founded as a non-profit entity near, but not linked to, a public hospital. With its personnel carefully selected and trained, ANT has maintained a core value of professionalism over its 30-year history. Also contributing to ANT's rapidly increasing popularity has been the development of a 'nice and friendly' environment for patients and families. This remains one of the most important points for patients attending day care; expressed values are to 'meet people' and 'get them out of the home' [12]. The services of volunteers, and initiatives such as the "Family Solidarity Project," reinforce this point of view. The number of patients has gradually increased over the years also because of better reporting procedures and through an increasing public awareness of the assistance project.

The choice for palliative care, but above all for death, away from home depends heavily on the culture and on local availability of services [15]. Patients' preferred place of death may influence strategic choices in the public health system, with important implications for palliative care delivery [16]. The costs and benefits of the two main models of palliative care, home care and hospice care, have been discussed for some time, with preferences for one or the other type of assistance depending in large measure on availability. In Italy, although many hospices now exist, differences between the North and South nonetheless lead to non-homogeneous access to hospice-based palliative care [17]. An extensive survey points out that in the year 2006, 78 hospices were active in northern Italy versus 10 in southern areas [18]. The high cost of hospice care may explain the difference, as southern Italian regions are poorer than northern ones. The presence of fewer hospices in the South than in the North could then be the result of local political choices, within discretional criteria of the Italian regional health care system, as determined by economic factors [19-21]. Other socio-cultural factors may explain differences between North and South of Italy. Such factors are the object of investigational studies. Up to now no definitive conclusion can be drawn on this issue. As a matter of fact, even when there is adequate hospice care to meet the demand, patients seem to chose home care. A recent survey in Italy found that approximately 1% of terminally ill cancer patients prefer to die in hospice, and no substantial differences were found between North and South of the country [22]. The fact that the foundation of a well-organized hospice network in the province of Bologna has not influenced the growth and operative size of ANT sustains the concept that the patient's preference is socially contingent rather than logistically determined [23-26].

These observations strongly suggest that, in Italy, the role of home-based care organizations should be carefully considered in planning palliative care programs.

Change in mean KPS at admission (higher/better functionality) and mean number of days of care (increasing) indicate a shift toward patients' accessing palliative care services at an earlier point in the disease/end-of-life trajectory. This trend may be the result of a mounting consciousness, among both doctors and patients, that early involvement in palliative care is beneficial. Among the benefits of early access to palliative care is continuity and coordination of care, and sufficient time to develop confidence in the organization to handle the progression of disease.

Over a 20-year period (1987-2007), the proportion of patients dying at home increased from 60% to 80%. Preferences for place of death can change with time and disease progression. A prospective study suggested that severity of functional impairments and symptom burden make patients reluctant to accept home care until absolutely necessary [26]. If patients and families are reassured by home-based assistance, this may increase the likelihood of preference for home death as disease progresses.

Across the years, ANT has increased the number of training courses for its staff and modified the organization of its team to include more nurses, physiotherapists, and psychologists. In practice, the OHH is increasingly developing a multidisciplinary approach characteristic of a palliative care team [27]. A culture increasingly supportive of palliative care and a progressively better knowledge about ANT services may have helped to spur this transition. Conferences on ANT and home-based palliative care have been a substantial part of ANT's long-standing educational program.

Quality of care was evaluated with a questionnaire completed by caregivers after the death of the patient; satisfaction with care was very high. However despite the fact that satisfaction is an important indicator of quality of care, these results should be considered with prudence because of the subjective variability in perception of care as opposed to more objective quality measures [28-31]. Similarly, satisfaction with care may be different and it may change during the period of care or after the death of the patient [32,33].

## Conclusions

The ANT model, born in Bologna, has demonstrated successful expansion and impact. The growth of OHHs in each part of Italy, measured by increasing numbers of patients served, indicates that the model is widely accepted and its concepts are well-understood in different social and cultural contexts. Practical experience thus sustaining its validity and its leading role as the currently best-accepted model of palliative care in our country. Analysis of ANT history suggests certain principal success factors. First, ANT identified a social need, and addressed it within the context of the local cultural and social environment. Second, the character of the organization and its approach to the patient and family facilitated its acceptance; attention to how staff enters people's homes has proven of fundamental importance to the rapport between providers of care and patients/families. Third, the organization possesses an internal solidarity which is manifested in its rigorous focus on two primary areas - education and service.

Finally, the changes in ANT observed over the 30 years reflect international trends in palliative care. We observed increasing uptake of the service across the region, with more patients referred earlier in their disease trajectory. Over time, as our service developed to meet more needs of the patients at home, the trend towards home death increased. As we developed our internal processes, we instituted evaluation measures and quality assurance programs. By chronicling our experience, we provide a snapshot of important trends in palliative care that are playing out not only in Italy, but worldwide.

## Competing interests

The authors declare that they have no competing interests.

## Authors' contributions

MC designed and developed the manuscript and participated in the collection of data. GB participated in the drafting of the manuscript. AA revised the final draft of the manuscript. VB was involved in collecting the data. RP participated in the drafting of the manuscript. FP participated in the drafting of the manuscript. All authors read and approved the final manuscript.

## Pre-publication history

The pre-publication history for this paper can be accessed here:

http://www.biomedcentral.com/1472-684X/9/12/prepub
